# Prognostic significance of laterality in lung neuroendocrine tumors

**DOI:** 10.1007/s12020-022-03015-w

**Published:** 2022-03-18

**Authors:** Anna La Salvia, Irene Persano, Alessandra Siciliani, Monica Verrico, Massimiliano Bassi, Roberta Modica, Alessandro Audisio, Isabella Zanata, Beatrice Trabalza Marinucci, Elena Trevisi, Giulia Puliani, Maria Rinzivillo, Elena Parlagreco, Roberto Baldelli, Tiziana Feola, Franz Sesti, Paola Razzore, Rossella Mazzilli, Massimiliano Mancini, Francesco Panzuto, Marco Volante, Elisa Giannetta, Carmen Romero, Marialuisa Appetecchia, Andrea Isidori, Federico Venuta, Maria Rosaria Ambrosio, Maria Chiara Zatelli, Mohsen Ibrahim, Annamaria Colao, Maria Pia Brizzi, Rocío García-Carbonero, Antongiulio Faggiano

**Affiliations:** 1 Department of Oncology, 12 de Octubre University Hospital, Madrid, Spain; 2 Department of Oncology, San Luigi Gonzaga Hospital, Orbassano, Italy; 3 Department of Thoracic Surgery, Sant’Andrea University Hospital, Rome, Italy; 4 Department of Radiological, Oncological, and Pathological Sciences, Sapienza University of Rome, Rome, Italy; 5 Department of Thoracic Surgery, Policlinico Umberto I, “Sapienza” University of Rome, Rome, Italy; 6 Department of Clinical Medicine and Surgery, University of Naples Federico II, Naples, Italy; 7 Department of Medical Sciences, Section of Endocrinology and Internal Medicine, University of Ferrara, Ferrara, Italy; 8 Oncological Endocrinology Unit, Regina Elena National Cancer Institute, Rome, Italy; 9 Department of Experimental Medicine, “Sapienza” University of Roma, Rome, Italy; 10 Digestive Disease Unit, ENETS Center of Excellence, Sant’Andrea University Hospital, Rome, Italy; 11 Endocrinology Unit, Department of Oncology and Medical Specialities, A.O. San Camillo-Forlanini, Rome, Italy; 12 Neuroendocrinology, Neuromed Institute, IRCCS, Pozzilli, Italy; 13 Endocrinology Unit, Mauriziano Hospital, Turin, Italy; 14 Endocrinology Unit, Department of Clinical and Molecular Medicine, Sant’Andrea Hospital, Sapienza University of Rome, ENETS Center of Excellence, Rome, Italy; 15 Division of Morphologic and Molecular, S. Andrea Hospital, Rome, Italy; 16 Department of Oncology, Pathology Unit of San Luigi Hospital, University of Turin, Orbassano, Turin, Italy; 17 Scientific Support, 12 de Octubre University Hospital, Madrid, Spain

**Keywords:** Lung neuroendocrine tumors, Tumor location, Ki67 index, Mitotic count, Necrosis, Prognostic factors.

## Abstract

**Purpose:**

Well-differentiated lung neuroendocrine tumors (Lu-NET) are classified as typical (TC) and atypical (AC) carcinoids, based on mitotic counts and necrosis. However, prognostic factors, other than tumor node metastasis (TNM) stage and the histopathological diagnosis, are still lacking. The current study is aimed to identify potential prognostic factors to better stratify lung NET, thus, improving patients’ treatment strategy and follow-up.

**Methods:**

A multicentric retrospective study, including 300 Lung NET, all surgically removed, from Italian and Spanish Institutions.

**Results:**

Median age 61 years (13–86), 37.7% were males, 25.0% were AC, 42.0% were located in the lung left parenchyma, 80.3% presented a TNM stage I-II. Mitotic count was ≥2 per 10 high-power field (HPF) in 24.7%, necrosis in 13.0%. Median overall survival (OS) was 46.1 months (0.6–323), median progression-free survival (PFS) was 36.0 months (0.3–323). Female sex correlated with a more indolent disease (T1; N0; lower Ki67; lower mitotic count and the absence of necrosis). Left-sided primary tumors were associated with higher mitotic count and necrosis. At Cox-multivariate regression model, age, left-sided tumors, nodal (N) positive status and the diagnosis of AC resulted independent negative prognostic factors for PFS and OS.

**Conclusions:**

This study highlights that laterality is an independent prognostic factors in Lu-NETs, with left tumors being less frequent but showing a worse prognosis than right ones. A wider spectrum of clinical and pathological prognostic factors, including TNM stage, age and laterality is suggested. These parameters could help clinicians to personalize the management of Lu-NET.

## Introduction

Neuroendocrine neoplasms of the lung (lung NENs) are classified according to 2021 WHO classification [1] into four major groups, depending on morphology, mitotic count, and necrosis.

Well-differentiated (WD) lung neuroendocrine tumors (Lu-NET) are categorized in typical (TC) and atypical (AC) carcinoids [1]. The differences between these two entities are defined according to mitotic count and the evaluation of occurrence and extent of necrosis (with mitotic count <2 per 10 high-power field (HPF) and absence of necrosis for the diagnosis of TC and mitotic count ≥2 per 10 HPF and/or the presence of necrosis for the diagnosis of AC). Poorly differentiated (PD) tumors are sub-classified into small cells and large cells lung neuroendocrine carcinomas (SCLCs and LCNECs), depending on cellular size. PD lung NEN are characterized by high mitotic count (>10 per 10 HPF), the presence of necrosis and are associated with an aggressive behavior and a dismal prognosis [2].

To date, the most relevant prognostic factor for Lu-NET is the histological diagnosis of TC or AC [3]. TC are considered indolent tumors with low recurrence rates and favorable prognosis, whereas AC are a more heterogeneous group in terms of clinical presentation and course, response to therapies and survival [4]. Surgery is the treatment of choice for both TC and AC, with a 5- and 10-year survival rate greater than 90% for TC and a 5-year survival rate ranging from 56 to 87% in AC [4, 5].

Traditionally, other factors have been established for their prognostic value, such as tumor node metastasis (TNM) stage [6] and especially nodal status [7]. However, TNM staging itself has demonstrated to be insufficient to correctly stratify Lu-NET. Thereby, the need to integrate pathological and molecular features to TNM staging has been suggested by several recent works [8, 9].

Many efforts have been made to deepen the molecular landscape of Lu-NETs. In the context of sporadic Lu-NETs, genome sequencing analyses have illustrated that chromatin-remodeling is the foremost as often as possible deregulated pathway, and *MEN1* [10], *PSIP1* and *ARID1A* the most commonly mutated genes [11]. Another study has suggested a potentially relevant role for other genes, as *BCL2* and *BCL2/BAX*, associated with anti-apoptotic activity, as independent prognostic parameters in Lu-NETs [12].

Interestingly, some studies have showed how the genetic alterations of TC differ from those of AC [13, 14]. A study conducted with next generation sequencing (NGS) on 148 lung NENs, including 88 TC and AC, demonstrated that MEN1 alterations were almost exclusive to WD Lu-NETs, whereas alterations of TP53 and RB1 genes were significantly enriched in SCLC and LCNECs [15]. However, in this analysis AC showed a hybrid pattern, whereby gains of *TERT*, *SDHA*, *RICTOR*, *PIK3CA*, *MYCL*, and *SRC* were found at rates similar to those in PD lung NENs. More recently, a comprehensive molecular characterization of a large population of Lu-NET has been carried out. In this study, the authors, through machine learning and multi-omics factor analysis, have identified that according to tumor genomic profile a group of AC should be considered similar to PD, high-grade LCNEC [16]. This group has been defined as “supracarcinoids” and has resulted associated with a dismal prognosis.

In the same direction, increasing evidences are arising about an intrinsic heterogeneity within Lu-NET. Beyond the morphological classification, the Ki67 index has emerged as a key feature to stratify Lu-NET [17, 18], as also observed in gastro-entero-pancreatic (GEP) NET [19, 20]. Lu-NET with a Ki67 > 20% have been associated with a significantly worse outcome than those with Ki67 ≤ 20% [21, 22].

Moreover, immunostaining for neuroendocrine markers such as chromogranin A, synaptophysin, or transcriptional thyroid factor 1 (TTF-1) have been identified in pulmonary NETs [23]. Unfortunately, a prognostic value for these tissue biomarkers has not been confirmed [2].

Furthermore, a prognostic role for different clinical factors, as age, sex, primary tumor location (central vs. peripheral, left vs. right lung), or pathological features as tumor grade, Ki67 index or mitotic count itself has not been established, so far [24, 25].

In the current study, we retrospectively analyze the features of 300 Lu-NET, from Italian and Spanish Institutions, to identify potential prognostic factors to better stratify Lu-NET patients and, in this way, improving patients’ treatment strategy and follow-up.

## Materials and methods

A multicentric retrospective study, including 300 Lu-NET, all surgically removed, from 8 Italian (San Luigi Gonzaga Hospital, Orbassano; Policlinico Umberto I, “Sapienza” University of Rome; European Neuroendocrine Tumor Society (ENETS) Center of Excellence, Sant’Andrea University Hospital, Rome; ENETS Center of Excellence, University of Naples Federico II, Naples; Regina Elena National Cancer Institute, Rome, University of Ferrara, Ferrara; San Camillo Forlanini Hospital, Rome and Mauriziano Hospital, Turin) and 1 Spanish (University Hospital 12 de Octubre, Madrid) NET-referral Institutions.

### Patients population

We retrospectively included all consecutive patients with a histologically confirmed diagnosis of WD Lu-NET (codified as TC or AC, according to 2015 WHO classification) and surgical removal of primary tumor. Extra-pulmonary NET, non-operated NET and PD NEN were excluded.

**Fig. 1:**
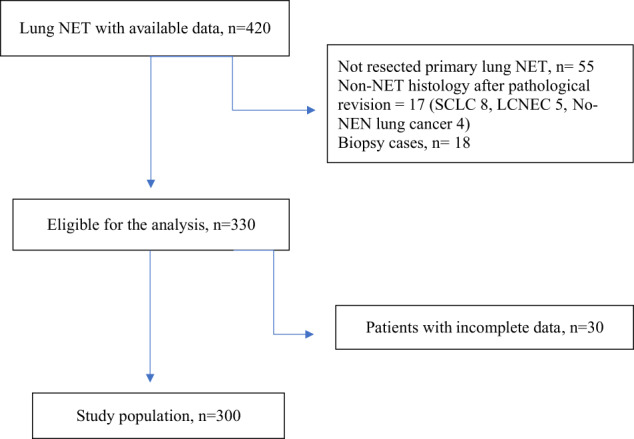
Patients’ selection flowchart

Based on these inclusion and exclusion criteria, we enrolled 420 patients, diagnosed and treated at one of the selected Italian or Spanish Institutions. After excluding patients with no resected primary tumor, PD neuroendocrine carcinomas and with no-neuroendocrine histology, patients that underwent surgery for the purpose of biopsy, 330 patients were eligible for analysis. Furthermore, after excluding patients with insufficient data, a total of 300 patients diagnosed with Lu-NET constituted the study population. Patient selection schema is summarized in Fig. 1.

### Clinical and pathological variables considered

Detailed patients’ characteristics were collected, including age, sex, and smoking habit. Tumoral features, comprehending tumor location central or peripheral, the location of the primary tumor in left or right lung parenchyma, TNM stage, “T” parameter itself (size of the primary tumor), “N” parameter itself (nodal involvement by the tumor), 18-FDG-PET-CT and 68-Gallium-DOTATOC PET-CT positivity, date of diagnosis and date of surgery were gathered. All relevant histopathological features as mitotic count, necrosis, Ki67 index, grading and immunohistochemical (IHC) evaluation of Chromogranin A, Synaptophysin and TTF-1 were collected. Progression-free survival (PFS) and overall survival (OS) were gathered.

### Statistical analysis

Categorical variables are expressed as number (percentage). Continuous variables are presented as median (range). PFS and OS were estimated using the Kaplan–Meier method; PFS was calculated from the date of surgery to the date of first progression according to RECIST criteria v1.1, or disease-related death for PFS; and OS to the date of death or last follow-up. We performed Chi square test to identify associations between different variables. A *p*-value of <0.05 was considered statistically significant. Univariate analyses were performed using Cox regression test for each variable of interest, to detect the impact on PFS and OS. For continuous parameters, the threshold was defined as the median value of the population. Multivariate analyses using a Cox proportional hazards regression analysis were performed to identify factors independently associated with prognosis. The results from the survival analyses are presented with the effect estimates, hazard ratios (HR), and 95% confidence interval [95%CI]. All statistical analyses were performed using IBM-SPSS version 25 (IBM Corporation, New-York, United States of America).

## Results

### Patients characteristics

Three-hundred patients with surgically removed Lu-NET were included in the study. The median age was 61 years (range from 13–86 years), the sex was male in 113 cases (37.7%). Primary tumor side was left in 126 cases (42.0%); AC were 44 (58.6%) left-sided and 31 right-sided. TNM stage at diagnosis was stage I in 171 cases (57.0%) and 65 were N + (21.7%).

Two-hundred ninety-three patients (97.7%) had a complete surgical removal of the primary tumor. The type of surgical resection was lobectomy in 194 (64.7%) of cases, pneumonectomy in 12 (4.0%), bilobectomy in 13 (4.3%), sleeve resection in 10 (3.3%), segmental resection in 13 (4.3%), wedge resection in 27 (9.0%), and other types of surgical resections in 9 cases (3.0%).

The mitotic count was ≥2 per 10 HPF in 74 cases (24.7%), the necrosis was confirmed in 39 cases (13.0%). Ki67 was 1–2% in 143 (47.7%), 3–19% in 80 (26.7%), 20% in 1 case (0.3%) and >20% in 9 cases (3.0%). The mOS was 46.1 months (0.6–323) and the mPFS was 36.0 months (0.3–323).

**Fig. 2:**
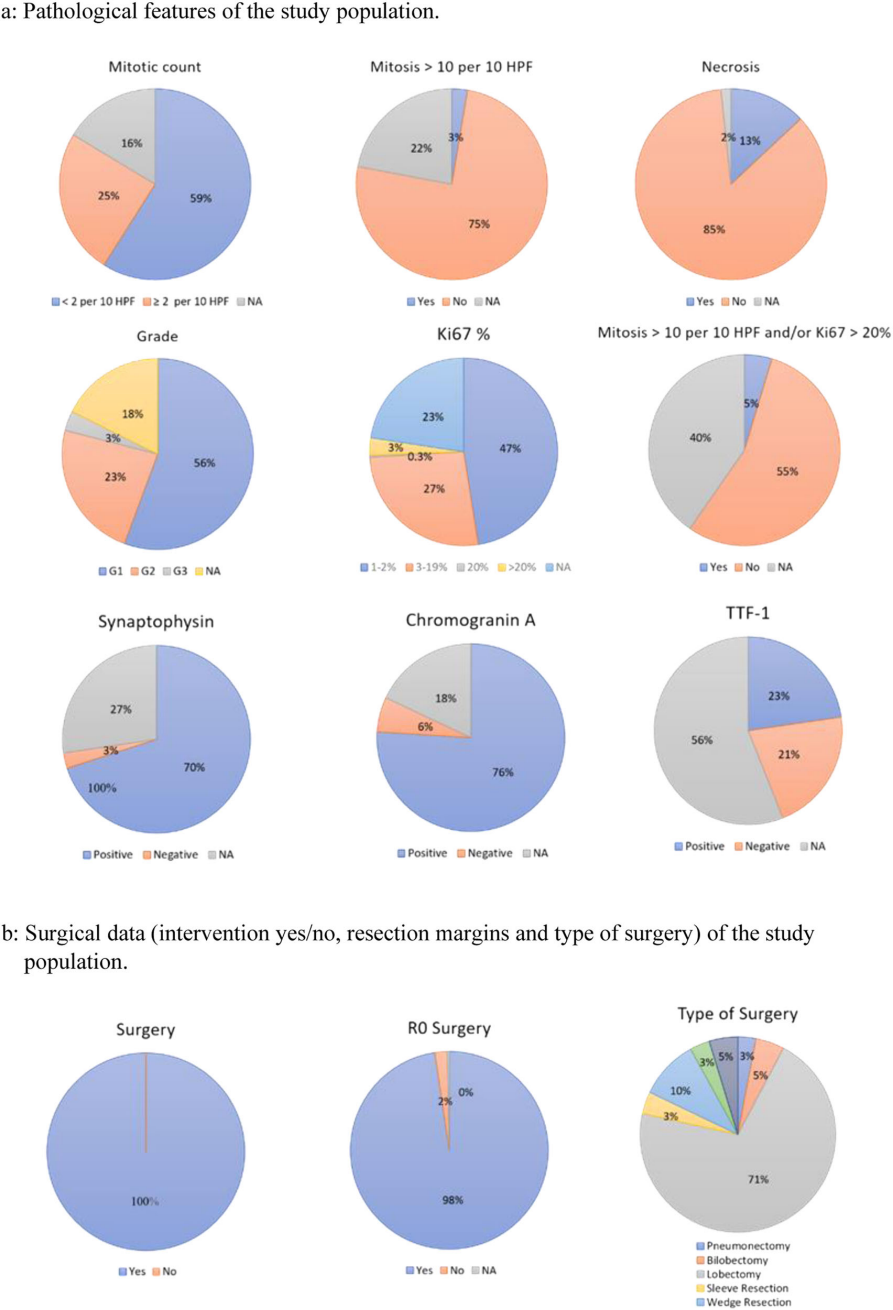
**a** Pathological features of the study population. **b** Surgical data (intervention yes/no, resection margins and type of surgery) of the study population

Patients’ clinical characteristics are summarized in Table 1. Pathological features and surgical data (intervention yes/no, resection margins and type of surgery) of the study population are detailed in Fig. 2a, b, respectively.

**Table 1:** Clinical characteristics of the study population

Characteristic	*N* = 300 (100%)
Sex	
Male	113 (37.7%)
Female	187 (62.3%)
Median age	
	61 years (13–86)
Smoke	
Yes	106 (35.3%)
No	142 (47.3%)
NA	52 (17.3%)
Tumor location	
Peripheral	112 (37.3%)
Central	186 (62.0%)
NA	2 (0.7%)
Tumor side (lung parenchyma)	
Left	126 (42.0%)
Right	172 (57.3%)
NA	2 (0.7%)
Diagnosis	
Typical carcinoid	225 (75.0%)
Atypical carcinoid	75 (25.0%)
Stage	
I	171 (57.0%)
II	70 (23.3%)
III	22 (7.3%)
IV	12 (4.0%)
NA	25 (8.3%)
T	
T1	177 (59.0%)
T2	78 (26.0%)
T3	16 (5.3%)
T4	7 (2.3%)
NA	22 (7.3%)
Nodal status	
N0	202 (67.3%)
N+	65 (21.7%)
NA	33 (11.0%)
18-FDG PET positivity	
Yes	130 (43.3%)
No	72 (24.0%)
NA	98 (32.7%)
68-Gallium PET/Octreoscan positivity	
Yes	42 (14.0%)
No	22 (7.3%)
NA	236 (78.7%)
Surgery	
Yes	300 (100%)
No	0 (0%)
R0 Surgery	
Yes	293 (97.7%)
No	6 (2.0%)
NA	1 (0.3%)
Type of surgery	
Pneumonectomy	12 (4.0%)
Bilobectomy	13 (4.3%)
Lobectomy	194 (64.7%)
Sleeve resection	10 (3.3%)
Segmental resection	13 (4.3%)
Wedge resection	27 (9.0%)
Other	9 (3.0%)
Progression	
Yes	62 (20.7%)
No	227 (75.7%)
NA	11 (3.7%)
Alive	
Yes	260 (86.7%)
No	30 (10.0%)
NA	10 (3.3%)
Median OS	
	46.1 months (0.6–323)
Median PFS	
	36.0 months (0.3–323)

*HPF* high-power field, *NA* not available, *OS* overall survival, *PFS* progression-free survival

**Fig. 3:**
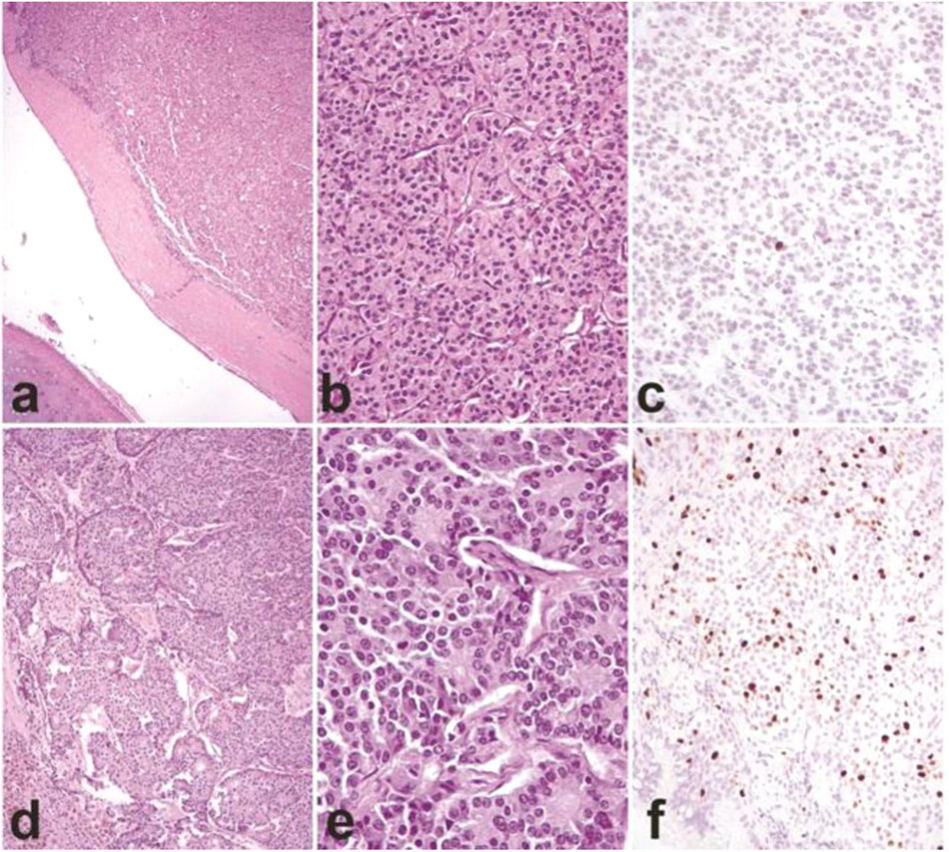
**a**–**c** Typical lung carcinoid. Endobronchial, right-sided, lesion (**a**) showing an organoid architecture (**b**) and low Ki-67 proliferative index (**c**). **d**–**f** Atypical carcinoid lung carcinoid. Peripheral, left-sided, lesion (**d**) with well-differentiated morphology (**e**) and high Ki-67 proliferative index (exceeding 20%) (**f**) **a**, **d**: original magnification x100; **b**, **c**, **f**: original magnification x200; **e**: original magnification x400

The pathological findings of two illustrative cases are depicted in Fig. 3.

#### Correlation with key variables: sex and primary tumor side

In Table 2, we report the associations between tumor side and patients’ sex, and the other variables determined by Chi square test.

**Table 2:** Associations between tumor side (left or right lung) and sex (male or female) and relevant clinico-pathological variables

	Side (left vs. right)	Sex (male vs. female)
Smoke (yes vs. no)	NS	*p* = 0.002
Location (peripheral vs. central)	NS	*p* = 0.025
T2-3-4 vs. T1	NS	*p* = 0.004
N+ vs. N0	NS	*p* < 0.0001
Mitotic count (≥or < 2 per 10 HPF)	*p* = 0.039	*p* = 0.001
Necrosis (yes vs. no)	*p* < 0.0001	*p* = 0.008
Ki67 (> or < 20%)	NS	*p* = 0.002
Grade (3 vs. 1–2)	NS	*p* < 0.0001

*HP*F high-power field, *NS* not significant. *p-*values have been considered as significant if <0.05

Sex was associated with smoking habit (*p* = 0.002), tumor location (*p* = 0.025), T (*p* = 0.004), N (*p* < 0.0001), grade (*p* < 0.0001), Ki67 (*p* = 0.002), mitotic count (*p* = 0.001), and necrosis (*p* = 0.008). Female sex correlated with a more indolent disease (T1 vs. T2-T3-T4; N0 vs. N positive; G1-2 vs. G3, Ki67 of 1–2%, mitotic count <2 per 10 HPF vs. ≥2 per 10 HPF and the absence of necrosis) and no smoking habit. Conversely, male sex was associated with a more aggressive disease (8/9 patients with Ki67 > 20% were males, as well as 9/10 patients with G3).

Primary tumor side was associated with mitotic count (*p* = 0.039) and the presence of necrosis (*p* < 0.0001). Right primary tumor side presented at diagnosis in 60.1% of cases with mitotic count <2 per 10 HPF. Conversely, the presence of necrosis was confirmed in 74.3% of cases in left-sided primary tumors.

The associations with patients’ age, peripheral vs. central location of the primary tumor and necrosis are detailed in the Supplementary materials.

#### Correlation with type of surgical resection

We also assessed the associations between the type of surgery and the other variables determined by Chi square test. The type of surgical removal was associated with primary tumor location central vs. peripheral (*p* < 0.001), with lobectomy performed in 130 central lesions (of 193 cases that underwent to lobectomy). All cases (*n* = 10) treated with sleeve resection were centrally located, whereas 20 of 16 cases who received a wedge resection presented a peripheral location. Type of surgery resulted associated also with T (*p* < 0.001), N (*p* = 0.001) and stage at diagnosis (*p* < 0.001). One-hundred sixty-nine of 186 patients that underwent to lobectomy presented a TNM stage I or II. Lobectomy, segmental, and wedge resection correlated with TC diagnosis (*p* = 0.008). Finally, lobectomy was associated with a mitotic count <2 per 10 HPF (*p* = 0.017) and to the absence of necrosis (*p* = 0.002).

### Prognostic impact on PFS and OS

#### Cox-univariate regression model

We detected a significant impact on patients’ outcome for sex, with male sex associated with dismal PFS (*p* < 0.0001) and OS (*p* < 0.0001) and for age, with a worse PFS for patient older than median age value (*p* = 0.038) and a worse OS for progressively higher age value (considered as continue variable, *p* = 0.012). A relevant impact for primary tumor side was found, with a negative prognostic impact for left-sided tumors (PFS *p* = 0.007, OS *p* = 0.014). Regarding the TNM stage at diagnosis, T2-3-4 vs. T1 (*p* < 0.0001, *p* = 0.011), N+ vs. N0 (*p* < 0.0001, *p* < 0.0001) and stage II-III-IV vs. stage I (*p* < 0.0001, *p* < 0.0001), correlated with shorter PFS and OS, respectively. Mitotic count ≥2 per 10 HPF vs. lower mitotic rate (*p* < 0.0001, *p* = 0.002), tumor grading (with G3 vs. G1-2: *p* < 0.0001, *p* < 0.0001), a Ki67 > 20% vs. lower Ki67 value (*p* = 0.001, *p* < 0.0001), the presence of necrosis vs. its absence (*p* < 0.0001, *p* = 0.003) and the diagnosis of AC vs. the diagnosis of TC (*p* < 0.0001, *p* < 0.0001), correlated with shorter PFS and OS. The positive immunostaining for Chromogranin A was associated with better OS (*p* = 0.022). Mitotic count >10 per 10 HPF (*p* < 0.0001) correlated with shorter PFS. Other variables, such as smoking habit, the type of surgical resection or primary tumor location central vs. peripheral were not significant in terms of patients’ outcomes.

**Fig. 4:**
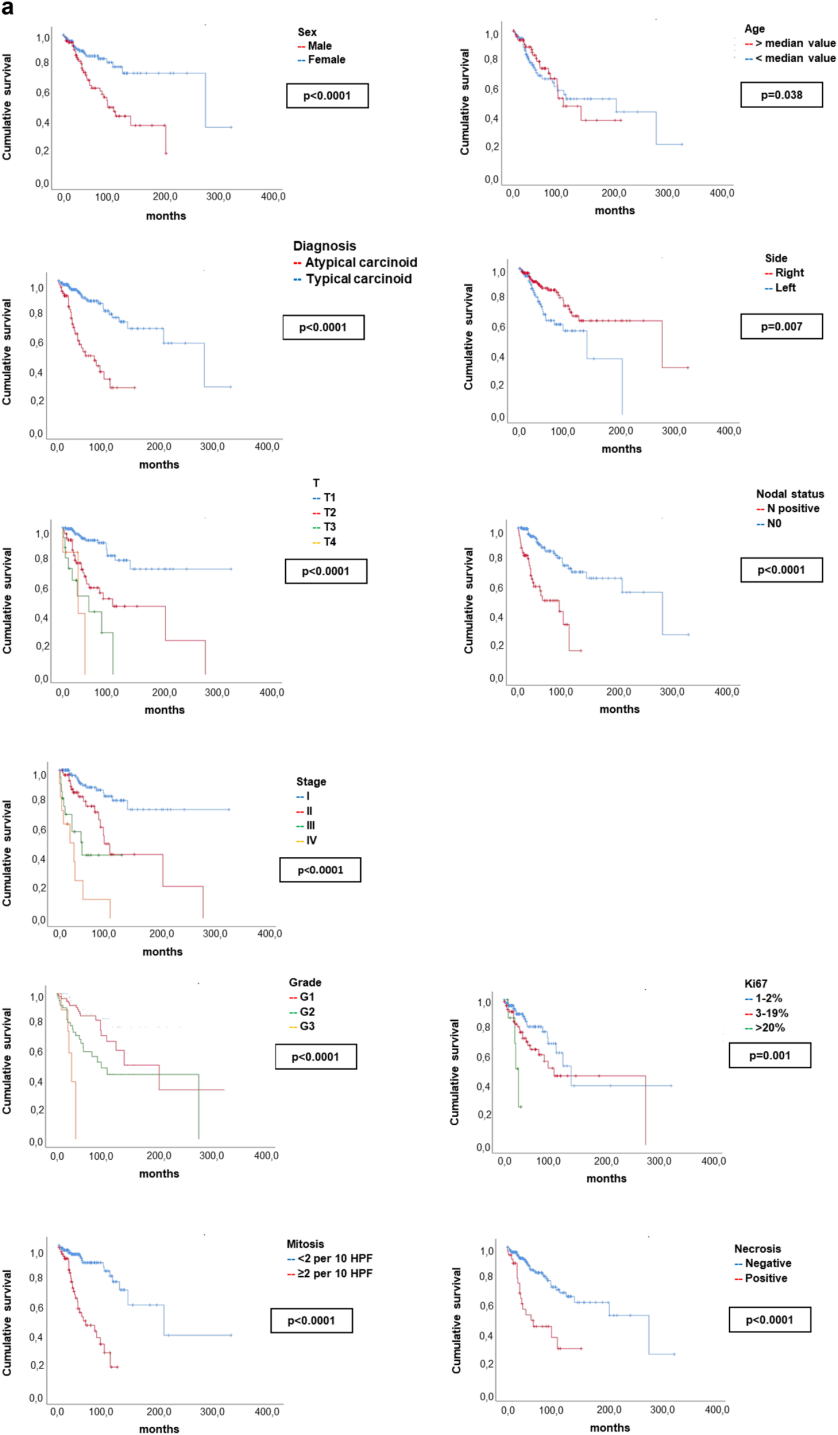

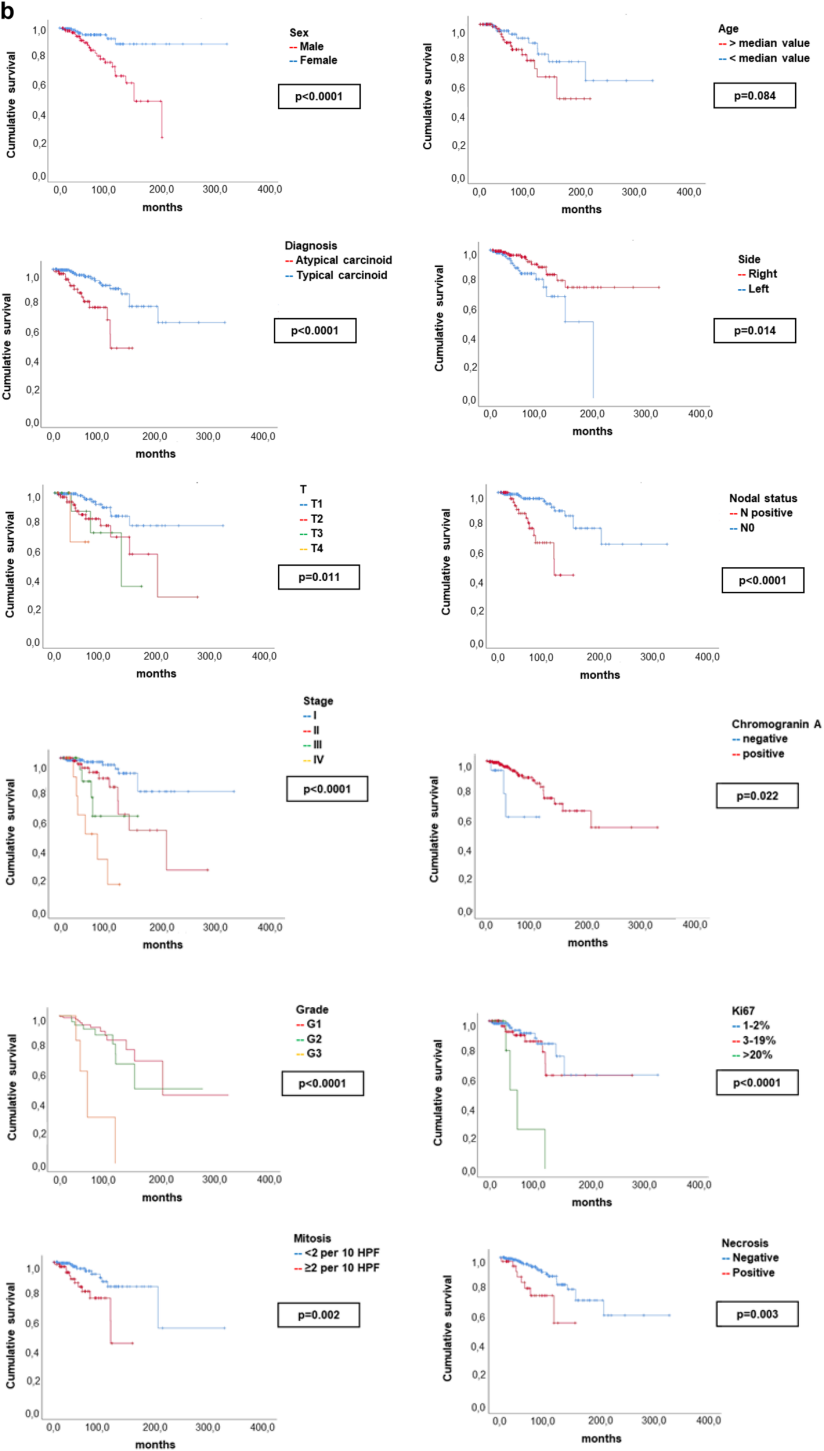
**a** Kaplan–Meier survival curves of the clinic-pathological variables with significant impact on patient’s survival in terms of PFS. *p*-values have been considered as significant if <0.05 and are reported according to the corresponding univariant Cox regression analysis. **b** Kaplan–Meier survival curves of the clinic-pathological variables with significant impact on patient’s survival in terms of OS. *p*-values have been considered as significant if <0.05 and are reported according to the corresponding univariant Cox regression analysis

Survival curves in terms of PFS and OS for the variables with significant impact on survival are reported in Fig. 4a (PFS: clinical and pathological variables) and Fig. 4b (OS: clinical and pathological variables). Additionally, we have explored the survival curves for mitotic count >10 per 10 HPF and Ki67 > 20%. These survival curves are depicted in the Supplementary materials.

#### Cox-multivariate regression analysis

At Cox-multivariate regression analysis, a progressively age value (considered as continue variable) *p* = 0.023, HR: 1.043 (95% CI, 1.006–1.082), tumor side (left vs. right) *p* = 0.043, HR: 3.033 (95% CI, 1.034–8.893) and N+ vs. N0 *p* = 0.003, HR: 6.481 HR (95% CI, 1.878–22.374) resulted independent negative prognostic factors for OS.

Additionally, a progressively age value *p* = 0.027, HR: 1.025 HR (95% CI, 1.003–1.047), tumor side (left vs. right) *p* = 0.071, HR: 1.823 (95% CI, 0.951–3.494), T 2-3-4 vs. T1 *p* = 0.000, HR: 2.064 (95% CI, 1.469–2.899) and N+ vs. N0 *p* = 0.011, HR: 2.598 (95% CI, 1.246.5.420) resulted independent negative prognostic factors in terms of PFS. The diagnosis of TC vs. AC was a protective factor for PFS (*p* = 0.002, HR: 0.281, 95% CI, 0.125–0.631).

The details of Cox-univariate and -multivariate regression analysis, in terms of OS and PFS, for variables with significant *p*-value are reported in Table 3(a) (OS) and (b) (PFS).

**Table 3:** (a) OS-multivariate analysis Cox regression model according to the following variables: age, sex, primary tumor side, T, N, the diagnosis of TC vs. AC and the presence of necrosis. (b) PFS-multivariate analysis Cox regression model according to the following variables: age, sex, primary tumor side, T, N, the diagnosis of TC vs. AC and the presence of necrosis

Variables	*p*	HR	HR 95.0% IC	
			Lowest	Highest
**(a)**				
Age	**0.023**	1.043	1.006	1.082
Sex (female vs. male)	0.159	0.442	0.142	1.377
Tumor side (left vs. right)	**0.043**	3.033	1.034	8.893
T (2-3-4 vs. T1)	0.131	1.525	0.882	2.637
N (positive vs. negative)	**0.003**	6.481	1.878	22.374
Diagnosis (TC vs. AC)	0.212	0.466	0.140	1.546
Necrosis (positive vs. negative)	0.125	3.100	0.732	13.130
**(b)**				
Age	**0.027**	1.025	1.003	1.047
Sex (female vs. male)	0.971	0.988	0.501	1.947
Tumor side (left vs. right)	**0.071**	1.823	0.951	3.494
T (2-3-4 vs. T1)	**0.000**	2.064	1.469	2.899
N (positive vs. negative)	**0.011**	2.598	1.246	5.420
Diagnosis (TC vs. AC)	**0.002**	0.281	0.125	0.631
Necrosis (positive vs. negative)	0.397	1.416	0.634	3.165

*IC* confidence interval, *HPF* high-power field, *HR* hazard ratio. *p-*values have been considered as significant if <0.05

## Discussion

Lu-NETs, account for ~30% of all NETs, 8.8% of all lung NENs (where SCLC accounts for 80% and LCNEC for 11.2%) and about 1–2% of all cancers of pulmonary origin [26]. Lu-NETs, given to their WD morphology and low proliferation index in the majority of cases are associated with an indolent clinical course and a relatively good prognosis if compared with PD lung NEC and also to the other subtypes of no-neuroendocrine lung cancers (as adenocarcinoma and squamous cell carcinoma) [27].

However, despite the existence of established morphological and immunohistochemical criteria for histopathological diagnosis for TC and AC (according to 2021 WHO classification), there is a lack of universal consensus for prognostic factors that could be useful for clinicians to better stratify these patients and to optimize their treatment strategy [28]. Therefore, the identification and the validation of reproducible clinical and pathological prognostic factors represents an urgent unmet need.

### TNM stage, T, N status, and tumor morphology (AC vs. TC): a confirmation of validated prognostic factors for Lu-NETs

TNM cancer staging is one of the most relevant and established prognostic tools across several types of tumors. TNM system quantify the extent of the primary tumor (T), lymph nodes (N), and distant metastases (M), providing a stage grouping [29]. TNM stage groups are of crucial importance in oncology [30]. Worsening survival rates with increasing TNM status have been demonstrated in all cancers [31, 32].

In the largest series of NET [33] and NEC [34], that are data obtained from the Surveillance, Epidemiology, and End Results (SEER) program, TNM stage has been confirmed as a fundamental prognostic marker, independently from primary tumor origin.

Given to their low incidence, Lu-NETs do not have a tumor-specific staging system. However, since the 7th edition, the TNM classification for lung cancer has been used for TC and AC, providing a clear stratification of patients with a major impact on patients’ outcomes [35]. In our study, TNM staging has been confirmed as a well-established prognostic factor for survival. This data was consistent with literature data [36].

In this context, a relevant role is represented by nodal status [7]. In a recent retrospective study, including 3.335 Lu-NETs, a positive nodal status emerged as independent negative prognostic factor (HR: 2.3), both for TC and AC [37]. In our study, N+ was associated with worse outcomes with a significant impact on PFS and OS, *p* = 0.011, HR: 2.598 and *p* = 0.003, HR: 6.481, respectively.

To date, the diagnosis of TC vs. AC represents the major indicator to guide the clinicians in their management of these patients. TC are associated with a biologically more indolent disease with a significant better prognosis if compared to AC [38–40]. A reason could be found in the recurrence rates, accounting for 16.6% in AC and 4.9% in TC [41]. A retrospective analysis of 62 AC, demonstrated that after a complete surgically removal, recurrences were observed mostly within the first 5 years of follow-up, within bronchi, mediastinal nodes, the liver, and bones [42]. In our study, the diagnosis of TC vs. AC clearly depicts two different groups, with a well-defined gap in terms of patients’ outcomes. Notably, the diagnosis of AC was confirmed as an independent negative prognostic factor in terms of PFS.

### Sex and age: challenging clinical factors

In our analysis, male sex correlated with a more aggressive disease, with a significant impact on survival. This observation, that clearly is showed in Kaplan–Meier curves of PFS and OS (*p* < 0.0001 and *p* < 0.0001, respectively). However, its independent value was not confirmed in our multivariant Cox regression model. According to SEER data, female patients were more likely to have a primary NET located in the lungs respect to males [43]. This data is confirmed in our study, where 62.3% of the patients included were female. Notably, a sex difference between TC and AC in males and females has been reported in literature, with a higher percentage of AC between males [44]. Several works report a correlation between male sex and a worse prognosis for Lu-NETs. This data is coherent with what observed in the no-neuroendocrine counterpart of lung tumors [45]. Filosso et al. reported the male sex as a negative prognostic factor for Lu-NETs [46]. In the same direction, the data coming from a retrospective analysis of 293 Lu-NETs, where OS was influenced by male sex. A recent study confirmed sex as independent factor for OS [47]. In this analysis female patients presented better prognosis than male (HR = 0.604, 95% CI = 0.45–0.806). On the other hand, in the Cox regression univariate analysis of 108 Lu-NET patients, sex was not associated with patient survival [48]. Recent analyses [49] have reported clinically relevant differences between male and female in NET originating from other primary sites (i.e., pancreatic NET).

Additionally, our analysis showed a relevant role for patients’ age. Our multivariate model confirmed the independent value of age, associating a higher risk of progression and death for increasing value of this variable. A role for age as a prognostic factor for NET has been previously reported [50, 51]. Focusing on Lu-NET, a retrospective analysis including TC selected from the SEER database, allowed to develop a nomogram to predict the probability of 3- and 5-year OS [52]. In this model longer OS was associated with younger age (*p* < 0.0001). This data has been confirmed by other studies, as a retrospective analysis of 108 Lu-NET [53] and an Asiatic study, including 64 lung carcinoids [54].

Therefore, considering our results and the available literature data, we suggest that sex and age should be evaluated as potential useful prognostic factors for Lu-NETs. Perspective larger studies are encouraged to validate this observation.

### Old parameters and new openings: mitotic count, necrosis, and Ki67 value

According to 2021 WHO classification, Lu-NETs are divided in two entities (TC and AC) according to two parameters, the mitotic count and the presence/absence of necrosis. These criteria are largely different from the ones for the diagnosis of NET with other origin [55]. For extra-pulmonary (EP) NETs the proliferation activity is quantified trough Ki67 index, which together with morphology, allows the codification of a grade for each case. Therefore, EP-NETs are separated in three classes, with a progressively higher Ki67 value, named G1, G2, and G3 NETs.

For Lu-NETs, several attempts have been made to introduce tumor grading and Ki67 to obtain a uniform classification for all sites NETs [56]. Some data exists about the utility of Ki67 as a prognostic biomarker even for Lu-NETs [57]. However, to date this type of classification has not been validated yet.

In our study, the two traditional pathological features for Lu-NETs, the mitotic count and the necrosis, confirmed their value also as prognostic factors. A mitotic count ≥2 per 10 HPF and the presence of necrosis correlated with a dismal outcome (in terms of PFS *p* < 0.0001 both, and OS *p* = 0.002 and *p* = 0.03).

Notably, in our study, 8 (2.7%) patients had a mitotic count >10 per 10 HPF. This subgroup presented a significant worse prognosis if compared to patients with lower mitotic count. This observation is intriguing, due to the 2021 WHO classification criteria that consider the mitotic count cut-off for Lu-NETs, equal to a maximum of 10 mitosis for 10 HPF. Taking together these data, a more heterogeneous and complex scenario could be hypothesized, comprehending Lu-NET characterized by a WD morphology associated with a high mitotic rate and a poor outcome.

Additionally, in our analysis 9 (3.0%) cases were reported to have a Ki67 > 20%. Among them, 5 presented a mitotic count ≤10 per 10 HPF, 2 a mitotic count ≥10 per 10 HPF (one of 12 and one of 14 per 10 HPF), this data was missing in the remaining 2 cases. This population of patients correlated with dismal outcome, in terms of PFS and OS, *p* = 0.006 and *p* < 0.0001, respectively. The Ki67 in this subgroup presented a median value of 30% and a range from 25% to 80%. Therefore, the highest Ki67 value in our study was 80%. This data differs from previous evidences, which reported lower maximum Ki67 index (65% in the study by Rubino et al. [58], 62% in the study by Oka et al. [21], 37% in the study by Kasajima et al. [22]). Arising literature data suggest that Lu-NET with a Ki67 > 20% are associated with a worse clinical outcome, if compared to WD-Lu-NET with lower Ki67 index [19]. An example is the study carried out by Rubino, with mOS of 203 months in patients with Ki67 index ≤5%, 101 months in patients with Ki67 index of 6–20% and 53 months in patients with Ki67 > 20% (*p* = 0.002) [58].

Therefore, the value of Ki67 as well as the role of mitotic count higher than 10 per 10 HPF, still remains an open and challenging question for Lu-NETs. A more complete diagnosis, taking into account tumor heterogeneity, immunohistochemistry and genomic profile, could help to provide new weapons to better characterize and stratify Lu-NETs, in the era of personalized medicine.

### New perspectives: primary tumor side, left-sided vs. right-sided tumors

Laterality is a potential significant prognostic factor for lung cancer because of the heterogeneity between left and right lung, in terms of anatomy, hematic and lymphatic circulation, relationship to the surrounding organs. The importance of right-sided vs. left-sided tumors has been clearly demonstrated for colon cancer, which exhibits differences in incidence, pathogenesis, molecular pathways and outcome depending on the location of the tumor [59]. A prognostic difference between right and left cancer has been also observed for the renal cell carcinoma by analyzing the SEER database. The worse prognosis in left than right tumors was related to earlier tumor stage, lower tumor grade, lymph node and distant metastasis [60].

However, a role for the side of primary tumor in the context of lung cancers in general and specifically for Lu-NET has not been established, so far. Previous studies in the field of Lu-NETs have demonstrated that right-sided tumors are more common [61], in analogy to our population (in which 172 cases were right-sided, accounting for 57.3% of the included population). A recent study analyzed the prognostic relevance of laterality in 1465 patients with non-small cell lung cancer undergone pneumonectomy [62]. Although right-sided pneumonectomy after induction therapy was associated with a significantly higher perioperative mortality, the 5-year survival was similar bewteen tumor sides. Anyway, this previous observation is not significant for the present study, due to the very low rate of pneumonectomy performed in lung NETs.

In our study, a highly significant association between tumor side and the presence of necrosis was found. In 39 of 300 cases, focal or diffuse necrosis was observed. 29 of them (74.3%) were detected in left-sided tumors, whereas only 10 (25.6%) in right-sided primaries. We observed also a significant negative impact for left tumor side on patients’ prognosis at the univariant analysis (PFS, *p* = 0.007 and OS *p* = 0.014). Therefore, we confirmed left side as an independent negative prognostic factor in terms of PFS (*p* = 0.043, HR: 3.033 (95% CI, 1.034–8.893) and OS *p* = 0.071, HR: 1.823 (95% CI, 0.951–3.494) at the multivariate analysis. The distribution of AC was in 44 in the left parenchyma and in 31 cases in right parenchyma. Among them, 39 of 75 cases were positive for necrosis at the histopathological evaluation, whereas the remaining 36 cases were diagnosed as AC due to the mitotic count ≥2 per 10 HPF only. Thereby, the simple distribution of AC cases between left and right parenchyma is not sufficient to explain the notably higher presence of necrosis in left tumors.

The consistence of this association between necrosis and left Lu-NETs could provide the rationale for a differential expression of angiogenesis and hypoxia according to the primary side of the tumor (right-sided vs. left-sided) with potentially relevant implications for patients’ outcomes. Therefore, we believe that this association should be better evaluated in further original and prospective studies to determine if primary tumor side could represent a new prognostic marker for Lu-NETs. Moreover, a better knowledge of these tumors biology and angiogenesis could pave the way for a more personalized approach in the therapeutic algorithm of these tumors.

The current study presents some limitations. First of all, the retrospective nature of patients’ inclusion, the collection of patients’ data and of the analysis. A second limit could be represented by patients’ sample size, although relatively adequate if we consider that NENs are rare tumors and between them, Lu-NET account for about 25–30%. Another limit is the percentage of missing data above all for pathological variables, as IHC for TTF-1 or Ki67 value (missing in 168 and 68 cases, respectively).

## Conclusions

This study highlights that laterality could be included among the prognostic factors with potential clinical relevance in Lu-NETs. The traditionally validated prognostic factors as TNM stage, nodal status and tumor morphology also confirmed their prognostic role. Left lung NETs showed a significantly higher rate of tumor necrosis than right tumors, suggesting a molecular basis for the negative outcomes of the formers. This finding needs to be investigated more in deep to establish the pathogenic rationale of the left-right difference. Other potentially relevant prognostic factors, which need to be further investigated are proliferative index, male sex, and age.

## Supplementary Information

Supplementary materials

## Data Availability

All data generated or analyzed during this study are included in this article. Further enquiries can be directed to the corresponding author.
