# Supplementary material for: Prognostic significance of laterality in lung neuroendocrine tumors
Source: Endocrine. 2022 Mar 18;76(3):733–46. doi: 10.1007/s12020-022-03015-w (PMC9156515; doi:10.1007/s12020-022-03015-w)

Supplementary Materials

New prognostic frontiers for lung neuroendocrine tumors

Anna La Salvia[1], Irene Persano[2], Alessandra Siciliani[3], Monica Verrico[4], Massimiliano Bassi[5], Roberta Modica[6], Alessandro Audisio[2], Isabella Zanata[7], Beatrice Trabalza Marinucci[3], Elena Trevisi[2], Giulia Puliani[8,9], Maria Rinzivillo[10], Elena Parlagreco[2], Roberto Baldelli[11], Tiziana Feola[9,12], Franz Sesti[9], Paola Razzore[13], Rossella Mazzilli[14], Massimiliano Mancini[15], Francesco Panzuto[10], Marco Volante[16], Elisa Giannetta[9], Carmen Romero[17], Marialuisa Appetecchia[8], Andrea Isidori[9], Federico Venuta[5], Maria Rosaria Ambrosio[7], Maria Chiara Zatelli[7], Mohsen Ibrahim[3], Annamaria Colao[6], Maria Pia Brizzi[2], Rocío García-Carbonero[1], Antongiulio Faggiano[14].

1. Department of Oncology, 12 de Octubre University Hospital, Madrid, Spain.
2. Department of Oncology, San Luigi Gonzaga Hospital, Orbassano, Italy.
3. Department of Thoracic Surgery, Sant'Andrea University Hospital, Rome, Italy.
4. Department of Radiological, Oncological and Pathological Sciences, Sapienza University of Rome, Rome, Italy.
5. Department of Thoracic Surgery, Policlinico Umberto I, "Sapienza" University of Rome, Italy.
6. Department of Clinical Medicine and Surgery, University of Naples Federico II, Naples, Italy.
7. Department of Medical Sciences, Section of Endocrinology and Internal Medicine, University of Ferrara, Ferrara, Italy.
8. Oncological Endocrinology Unit, Regina Elena National Cancer Institute, Rome, Italy.
9. Department of Experimental Medicine, "Sapienza" University of Roma, Italy.
10. Digestive Disease Unit, ENETS Center of Excellence, Sant'Andrea University Hospital, Rome, Italy.
11. Endocrinology Unit, Department of Oncology and Medical Specialities, A.O. San Camillo-Forlanini, Rome, Italy.
12. Neuroendocrinology, Neuromed Institute, IRCCS, Pozzilli, Italy.
13. Endocrinology Unit, Mauriziano Hospital, Turin, Italy.
14. Endocrinology Unit, Department of Clinical and Molecular Medicine, Sant'Andrea Hospital, Sapienza University of Rome, ENETS Center of Excellence, Rome, Italy.
15. Division of Morphologic and Molecular, S. Andrea Hospital, Rome, Italy.
16. Department of Oncology, Pathology Unit of San Luigi Hospital, University of Turin, Orbassano, Turin, Italy.
17. Scientific support, 12 de Octubre University Hospital, Madrid, Spain.

Short title: New prognostic frontiers for lung N

Corresponding author

Anna La Salvia
Department of Oncology, 12 de Octubre University Hospital
Avenida de Cordoba, s/n, 28041, Madrid, Spain
Telephone: +34 602485105
Email: alasalvi@ucm.es

Supplementary results. Associations with age, peripheral versus central location of the primary tumor and necrosis

1. AGE

Case Processing Summary

	Cases					
	Valid		Missing		Total	
	N	Percent	N	Percent	N	Percent
Age * Smoke yes 1 no 0	165	55,0%	135	45,0%	300	100,0%
Age* Central 1 vs Periferic 0 primary lesion	200	66,7%	100	33,3%	300	100,0%
Age * 18 FDG PET scan Not done Positive 1 Negative 0	103	34,3%	197	65,7%	300	100,0%
Age* Octreoscan/68GalliumPET Not done NA Positive 1 Negative 0	63	21,0%	237	79,0%	300	100,0%
Age * Tcat	192	64,0%	108	36,0%	300	100,0%
Age * Npos1neg0	190	63,3%	110	36,7%	300	100,0%
Age * Stage	196	65,3%	104	34,7%	300	100,0%
Age * Ki67	165	55,0%	135	45,0%	300	100,0%
Age* Necrosis Not done NA Positive 1 Negative 0	199	66,3%	101	33,7%	300	100,0%
Age* Diagnosis Typical carcinoid 0 Atypical carcinoid 1	200	66,7%	100	33,3%	300	100,0%
Age * Typesurgery	178	59,3%	122	40,7%	300	100,0%

Count

		Smoke yes 1 no 0		
		0	1	Total
Age	< = MED	47	37	84
	>MED	55	26	81
Total		102	63	165

Chi-Square Tests

	Value	df	Asymptotic Significance (2-sided)	Exact Sig. (2-sided)	Exact Sig. (1-sided)
Pearson Chi-Square	2,494[a]	1	0,114		
Continuity Correction[b]	2,014	1	0,156		
Likelihood Ratio	2,504	1	0,114		
Fisher's Exact Test				0,149	0,078
Linear-by-Linear Association	2,479	1	0,115		
N of Valid Cases	165				

a. 0 cells (0,0%) have expected count less than 5. The minimum expected count is 30,93.

b. Computed only for a 2x2 table

Crosstab

Count

		Central 1 vs Periferic 0 primary lesion		
		0	1	Total
Age	< = MED	27	76	103
	>MED	44	53	97
Total		71	129	200

Chi-Square Tests

	Value	df	Asymptotic Significance (2-sided)	Exact Sig. (2-sided)	Exact Sig. (1-sided)
Pearson Chi-Square	7,998[a]	1	0,005		
Continuity Correction[b]	7,184	1	0,007		
Likelihood Ratio	8,053	1	0,005		
Fisher's Exact Test				0,005	0,004
Linear-by-Linear Association	7,958	1	0,005		
N of Valid Cases	200				

a. 0 cells (0,0%) have expected count less than 5. The minimum expected count is 34,43.

b. Computed only for a 2x2 table

Crosstab

Count

		18 FDG PET scan Not done Positive 1 Negative 0		
		0	1	Total
Age	< = MED	12	36	48
	>MED	14	41	55
Total		26	77	103

Chi-Square Tests

	Value	df	Asymptotic Significance (2-sided)	Exact Sig. (2-sided)	Exact Sig. (1-sided)
Pearson Chi-Square	0,003[a]	1	0,958		
Continuity Correction[b]	0,000	1	1,000		
Likelihood Ratio	0,003	1	0,958		
Fisher's Exact Test				1,000	0,570
Linear-by-Linear Association	0,003	1	0,958		
N of Valid Cases	103				

a. 0 cells (0,0%) have expected count less than 5. The minimum expected count is 12,12.

b. Computed only for a 2x2 table

Crosstab

Count

		Octreoscan/68GalliumPET Not done NA Positive 1 Negative 0		
		0	1	Total
Age	< = MED	14	24	38
	>MED	8	17	25
Total		22	41	63

Chi-Square Tests

	Value	df	Asymptotic Significance (2-sided)	Exact Sig. (2-sided)	Exact Sig. (1-sided)
Pearson Chi-Square	0,156[a]	1	0,693		
Continuity Correction[b]	0,015	1	0,901		
Likelihood Ratio	0,156	1	0,693		
Fisher's Exact Test				0,790	0,453
Linear-by-Linear Association	0,153	1	0,696		
N of Valid Cases	63				

a. 0 cells (0,0%) have expected count less than 5. The minimum expected count is 8,73.

b. Computed only for a 2x2 table

Crosstab

Count

		Tcat				
		1	2	3	4	Total
Age	< = MED	53	33	10	3	99
	>MED	63	23	4	3	93
Total		116	56	14	6	192

Chi-Square Tests

	Value	df	Asymptotic Significance (2-sided)
Pearson Chi-Square	5,037[a]	3	0,169
Likelihood Ratio	5,128	3	0,163
Linear-by-Linear Association	3,152	1	0,076
N of Valid Cases	192		

a. 2 cells (25,0%) have expected count less than 5. The minimum expected count is 2,91.

Crosstab

Count

		Npos1neg0		
		0	1	Total
Age	< = MED	68	31	99
	>MED	72	19	91
Total		140	50	190

Chi-Square Tests

	Value	df	Asymptotic Significance (2-sided)	Exact Sig. (2-sided)	Exact Sig. (1-sided)
Pearson Chi-Square	2,662[a]	1	0,103		
Continuity Correction[b]	2,151	1	0,142		
Likelihood Ratio	2,686	1	0,101		
Fisher's Exact Test				0,137	0,071
Linear-by-Linear Association	2,648	1	0,104		
N of Valid Cases	190				

a. 0 cells (0,0%) have expected count less than 5. The minimum expected count is 23,95.

b. Computed only for a 2x2 table

Crosstab

Count

		Stage				
		1	2	3	4	Total
Age	< = MED	51	33	8	7	99
	>MED	60	23	9	5	97
Total		111	56	17	12	196

Chi-Square Tests

	Value	df	Asymptotic Significance (2-sided)
Pearson Chi-Square	2,887[a]	3	0,409
Likelihood Ratio	2,899	3	0,407
Linear-by-Linear Association	1,065	1	0,302
N of Valid Cases	196		

a. 0 cells (0,0%) have expected count less than 5. The minimum expected count is 5,94.

Crosstab

Count

		Ki67			Total
		1	2	3	
Age	< = MED	49	35	4	88
	>MED	34	39	4	77
Total		83	74	8	165

Chi-Square Tests

	Value	df	Asymptotic Significance (2-sided)
Pearson Chi-Square	2,204[a]	2	0,332
Likelihood Ratio	2,208	2	0,332
Linear-by-Linear Association	1,754	1	0,185
N of Valid Cases	165		

a. 2 cells (33,3%) have expected count less than 5. The minimum expected count is 3,73.

Crosstab

Count

		Necrosis Not done NA Positive 1 Negative 0		
		0	1	Total
Age	< = MED	83	20	103
	>MED	82	14	96
Total		165	34	199

Chi-Square Tests

	Value	df	Asymptotic Significance (2-sided)	Exact Sig. (2-sided)	Exact Sig. (1-sided)
Pearson Chi-Square	0,820[a]	1	0,365		
Continuity Correction[b]	0,514	1	0,473		
Likelihood Ratio	0,824	1	0,364		
Fisher's Exact Test				0,452	0,237
Linear-by-Linear Association	0,816	1	0,366		
N of Valid Cases	199				

a. 0 cells (0,0%) have expected count less than 5. The minimum expected count is 16,40.

b. Computed only for a 2x2 table

Crosstab

Count

		Diagnosis Typical carcinoid 0 Atypical carcinoid 1		
		0	1	Total
Age	< = MED	67	36	103
	>MED	72	25	97
Total		139	61	200

Chi-Square Tests

	Value	df	Asymptotic Significance (2-sided)	Exact Sig. (2-sided)	Exact Sig. (1-sided)
Pearson Chi-Square	1,985[a]	1	0,159		
Continuity Correction[b]	1,576	1	0,209		
Likelihood Ratio	1,994	1	0,158		
Fisher's Exact Test				0,170	0,105
Linear-by-Linear Association	1,975	1	0,160		
N of Valid Cases	200				

a. 0 cells (0,0%) have expected count less than 5. The minimum expected count is 29,59.

b. Computed only for a 2x2 table

Count

		Typesurgery								
		pneumonectomy	bilobectomy	lobectomy	sleeve resection	segmental resection	wedge resection	extended resection	other	Total
Age	< = MED	7	8	62	2	3	5	4	1	92
	>MED	4	2	67	0	2	9	2	0	86
Total		11	10	129	2	5	14	6	1	178

Chi-Square Tests

	Value	df	Asymptotic Significance (2-sided)
Pearson Chi-Square	9,430[a]	7	0,223
Likelihood Ratio	10,874	7	0,144
Linear-by-Linear Association	0,245	1	0,620
N of Valid Cases	178		

a. 9 cells (56,3%) have expected count less than 5. The minimum expected count is 0,48.

2. TUMOR LOCATION: PERIPHERAL VERSUS CENTRAL

Case Processing Summary

	Cases					
	Valid		Missing		Total	
	N	Per cent	N	Per cent	N	Percent
Central 1 vs Periferic 0 primary lesion * Smoke yes 1 no 0	246	82,0 %	54	18,0 %	300	100,0%
Central 1 vs Periferic 0 primary lesion * 18 FDG PET scan Not done Positive 1 Negative 0	200	66,7 %	100	33,3 %	300	100,0%
Central 1 vs Periferic 0 primary lesion * Octreoscan/68Gallium PET Not done NA Positive 1 Negative 0	64	21,3 %	236	78,7 %	300	100,0%
Central 1 vs Periferic 0 primary lesion * Tcat	276	92,0 %	24	8,0 %	300	100,0%
Central 1 vs Periferic 0 primary lesion * Npos1neg0	265	88,3 %	35	11,7 %	300	100,0%
Central 1 vs Periferic 0 primary lesion * Stage	274	91,3 %	26	8,7 %	300	100,0%
Central 1 vs Periferic 0 primary lesion * Ki67	231	77,0 %	69	23,0 %	300	100,0%
Central 1 vs Periferic 0 primary lesion * Necrosis Not done NA Positive 1 Negative 0	293	97,7 %	7	2,3 %	300	100,0%
Central 1 vs Periferic 0 primary lesion * Diagnosis Typical carcinoid 0 Atypical carcinoid 1	298	99,3 %	2	0,7 %	300	100,0%
Central 1 vs Periferic 0 primary lesion * Typesurgery	276	92,0 %	24	8,0 %	300	100,0%
Central 1 vs Periferic 0 primary lesion * Age	200	66,7 %	100	33,3 %	300	100,0%

Crosstab

Count

		Smoke yes 1 no 0		Total
		0	1	
Central 1 vs Periferic 0 primary lesion	0	65	37	102
	1	77	67	144
Total		142	104	246

Chi-Square Tests

	Value	df	Asymptotic Significance (2-sided)	Exact Sig. (2-sided)	Exact Sig. (1-sided)
Pearson Chi-Square	2,572[a]	1	0,109		
Continuity Correction[b]	2,169	1	0,141		
Likelihood Ratio	2,587	1	0,108		
Fisher's Exact Test				0,118	0,070
Linear-by-Linear Association	2,562	1	0,109		
N of Valid Cases	246				

a. 0 cells (0,0%) have expected count less than 5. The minimum expected count is 43,12.

b. Computed only for a 2x2 table

Crosstab

Count

		18 FDG PET scan Not done Positive 1 Negative 0		Total
		0	1	
Central 1 vs Periferic 0 primary lesion	0	21	62	83
	1	51	66	117
Total		72	128	200

Chi-Square Tests

	Value	df	Asymptotic Significance (2-sided)	Exact Sig. (2-sided)	Exact Sig. (1-sided)
Pearson Chi-Square	7,049[a]	1	0,008		
Continuity Correction[b]	6,277	1	0,012		
Likelihood Ratio	7,206	1	0,007		
Fisher's Exact Test				0,011	0,006
Linear-by-Linear Association	7,013	1	0,008		
N of Valid Cases	200				

a. 0 cells (0,0%) have expected count less than 5. The minimum expected count is 29,88.

b. Computed only for a 2x2 table

Count

		Octreoscan/68GalliumPET Not done NA Positive 1 Negative 0		Total
		0	1	
Central 1 vs Periferic 0 primary lesion	0	9	13	22
	1	13	29	42
Total		22	42	64

Chi-Square Tests

	Value	df	Asymptotic Significance (2-sided)	Exact Sig. (2-sided)	Exact Sig. (1-sided)
Pearson Chi-Square	0,634[a]	1	0,426		
Continuity Correction[b]	0,270	1	0,603		
Likelihood Ratio	0,627	1	0,428		
Fisher's Exact Test				0,580	0,300
Linear-by-Linear Association	0,625	1	0,429		
N of Valid Cases	64				

a. 0 cells (0,0%) have expected count less than 5. The minimum expected count is 7,56.

b. Computed only for a 2x2 table

Crosstab

Count

		Tcat				Total
		1	2	3	4	
Central 1 vs Periferic 0 primary lesion	0	72	22	7	2	103
	1	103	56	9	5	173
Total		175	78	16	7	276

Chi-Square Tests

	Value	df	Asymptotic Significance (2-sided)
Pearson Chi-Square	4,375[a]	3	0,224
Likelihood Ratio	4,480	3	0,214
Linear-by-Linear Association	1,415	1	0,234
N of Valid Cases	276		

a. 2 cells (25,0%) have expected count less than 5. The minimum expected count is 2,61.

Crosstab

Count

		Npos1neg0		Total
		0	1	
Central 1 vs Periferic 0 primary lesion	0	70	27	97
	1	130	38	168
Total		200	65	265

Chi-Square Tests

	Value	df	Asymptotic Significance (2-sided)	Exact Sig. (2-sided)	Exact Sig. (1-sided)
Pearson Chi-Square	0,904[a]	1	0,342		
Continuity Correction[b]	0,644	1	0,422		
Likelihood Ratio	0,894	1	0,344		
Fisher's Exact Test				0,375	0,210
Linear-by-Linear Association	0,900	1	0,343		
N of Valid Cases	265				

a. 0 cells (0,0%) have expected count less than 5. The minimum expected count is 23,79.

b. Computed only for a 2x2 table

Crosstab

Count

		Stage				Total
		1	2	3	4	
Central 1 vs Periferic 0 primary lesion	0	62	21	10	6	99
	1	108	49	12	6	175
Total		170	70	22	12	274

Chi-Square Tests

	Value	df	Asymptotic Significance (2-sided)
Pearson Chi-Square	2,978[a]	3	0,395
Likelihood Ratio	2,946	3	0,400
Linear-by-Linear Association	0,542	1	0,462
N of Valid Cases	274		

a. 1 cells (12,5%) have expected count less than 5. The minimum expected count is 4,34.

Crosstab

Count

		Ki67			Total
		1	2	3	
Central 1 vs Periferic 0 primary lesion	0	59	23	2	84
	1	83	57	7	147
Total		142	80	9	231

Chi-Square Tests

	Value	df	Asymptotic Significance (2-sided)
Pearson Chi-Square	4,432[a]	2	0,109
Likelihood Ratio	4,536	2	0,104
Linear-by-Linear Association	4,312	1	0,038
N of Valid Cases	231		

a. 1 cells (16,7%) have expected count less than 5. The minimum expected count is 3,27.

Crosstab

Count

		Necrosis Not done NA Positive 1 Negative 0		Total
		0	1	
Central 1 vs Periferic 0 primary lesion	0	97	12	109
	1	157	27	184
Total		254	39	293

Chi-Square Tests

	Value	df	Asymptotic Significance (2-sided)	Exact Sig. (2-sided)	Exact Sig. (1-sided)
Pearson Chi-Square	0,797[a]	1	0,372		
Continuity Correction[b]	0,511	1	0,475		
Likelihood Ratio	0,815	1	0,367		
Fisher's Exact Test				0,477	0,239
Linear-by-Linear Association	0,794	1	0,373		
N of Valid Cases	293				

a. 0 cells (0,0%) have expected count less than 5. The minimum expected count is 14,51.

b. Computed only for a 2x2 table

Crosstab

Count

		Diagnosis Typical carcinoid 0 Atypical carcinoid 1		Total
		0	1	
Central 1 vs Periferic 0 primary lesion	0	87	25	112
	1	136	50	186
Total		223	75	298

Chi-Square Tests

	Value	df	Asymptotic Significance (2-sided)	Exact Sig. (2-sided)	Exact Sig. (1-sided)
Pearson Chi-Square	0,772[a]	1	0,380		
Continuity Correction[b]	0,549	1	0,459		
Likelihood Ratio	0,781	1	0,377		
Fisher's Exact Test				0,411	0,230
Linear-by-Linear Association	0,769	1	0,380		
N of Valid Cases	298				

a. 0 cells (0,0%) have expected count less than 5. The minimum expected count is 28,19.

b. Computed only for a 2x2 table

3. NECROSIS

Case Processing Summary

	Cases					
	Valid		Missing		Total	
	N	Percent	N	Percent	N	Percent
Necrosis Not done NA Positive 1 Negative 0 * Smoke yes 1 no 0	245	81,7%	55	18,3%	300	100,0%
Necrosis Not done NA Positive 1 Negative 0 * 18 FDG PET scan Not done Positive 1 Negative 0	198	66,0%	102	34,0%	300	100,0%
Necrosis Not done NA Positive 1 Negative 0 * Octreoscan/68GalliumPET Not done NA Positive 1 Negative 0	64	21,3%	236	78,7%	300	100,0%
Necrosis Not done NA Positive 1 Negative 0 * Tcat	274	91,3%	26	8,7%	300	100,0%
Necrosis Not done NA Positive 1 Negative 0 * Npos1neg0	264	88,0%	36	12,0%	300	100,0%
Necrosis Not done NA Positive 1 Negative 0 * Stage at diagnosis	295	98,3%	5	1,7%	300	100,0%
Necrosis Not done NA Positive 1 Negative 0 * Gruppoki67	229	76,3%	71	23,7%	300	100,0%

Crosstab

Count

		Smoke yes 1 no 0		Total
		0	1	
Necrosis Not done NA Positive 1 Negative 0	0	126	81	207
	1	16	22	38
Total		142	103	245

Chi-Square Tests

	Value	df	Asymptotic Significance (2-sided)	Exact Sig. (2-sided)	Exact Sig. (1-sided)
Pearson Chi-Square	4,639[a]	1	0,031		
Continuity Correction[b]	3,901	1	0,048		
Likelihood Ratio	4,578	1	0,032		
Fisher's Exact Test				0,048	0,025
Linear-by-Linear Association	4,620	1	0,032		
N of Valid Cases	245				

a. 0 cells (0,0%) have expected count less than 5. The minimum expected count is 15,98.

b. Computed only for a 2x2 table

Crosstab

Count

		18 FDG PET scan Not done Positive 1 Negative 0		Total
		0	1	
Necrosis Not done NA Positive 1 Negative 0	0	56	116	172
	1	12	14	26
Total		68	130	198

Chi-Square Tests

	Value	df	Asymptotic Significance (2-sided)	Exact Sig. (2-sided)	Exact Sig. (1-sided)
Pearson Chi-Square	1,851[a]	1	0,174		
Continuity Correction[b]	1,298	1	0,255		
Likelihood Ratio	1,786	1	0,181		
Fisher's Exact Test				0,189	0,128
Linear-by-Linear Association	1,842	1	0,175		
N of Valid Cases	198				

a. 0 cells (0,0%) have expected count less than 5. The minimum expected count is 8,93.

b. Computed only for a 2x2 table

Crosstab

Count

		Octreoscan/68GalliumPET Not done NA Positive 1 Negative 0		Total
		0	1	
Necrosis Not done NA Positive 1 Negative 0	0	18	32	50
	1	4	10	14
Total		22	42	64

Chi-Square Tests

	Value	df	Asymptotic Significance (2-sided)	Exact Sig. (2-sided)	Exact Sig. (1-sided)
Pearson Chi-Square	0,268[a]	1	0,605		
Continuity Correction[b]	0,040	1	0,842		
Likelihood Ratio	0,274	1	0,601		
Fisher's Exact Test				0,755	0,429
Linear-by-Linear Association	0,263	1	0,608		
N of Valid Cases	64				

a. 1 cells (25,0%) have expected count less than 5. The minimum expected count is 4,81.

b. Computed only for a 2x2 table

Crosstab

Count

		Tcat				Total
		1	2	3	4	
Necrosis Not done NA Positive 1 Negative 0	0	168	56	9	3	236
	1	7	20	7	4	38
Total		175	76	16	7	274

Chi-Square Tests

	Value	df	Asymptotic Significance (2-sided)
Pearson Chi-Square	47,059[a]	3	0,000
Likelihood Ratio	42,735	3	0,000
Linear-by-Linear Association	46,326	1	0,000
N of Valid Cases	274		

a. 2 cells (25,0%) have expected count less than 5. The minimum expected count is ,97.

Crosstab

Count

		Npos1neg0 0	1	Total
Necrosis Not done NA Positive 1 Negative 0	0	180	47	227
	1	19	18	37
Total		199	65	264

Chi-Square Tests

	Value	df	Asymptotic Significance (2-sided)	Exact Sig. (2-sided)	Exact Sig. (1-sided)
Pearson Chi-Square	13,386[a]	1	,000		
Continuity Correction[b]	11,922	1	,001		
Likelihood Ratio	11,881	1	,001		
Fisher's Exact Test				,001	,001
Linear-by-Linear Association	13,335	1	,000		
N of Valid Cases	264				

a. 0 cells (0,0%) have expected count less than 5. The minimum expected count is 9,11.

b. Computed only for a 2x2 table

Crosstab

Count

		Gruppoki67 1	2	3	Total
Necrosis Not done NA Positive 1 Negative 0	0	135	54	5	194
	1	7	25	3	35
Total		142	79	8	229

Chi-Square Tests

	Value	df	Asymptotic Significance (2-sided)
Pearson Chi-Square	31,141[a]	2	,000
Likelihood Ratio	30,850	2	,000
Linear-by-Linear Association	29,173	1	,000
N of Valid Cases	229		

a. 1 cells (16,7%) have expected count less than 5. The minimum expected count is 1,22.

Crosstab

Count

		Diagnosis Typical carcinoid 0 Atypical carcinoid 1		Total
		0	1	
Necrosis Not done NA Positive 1 Negative 0	0	225	31	256
	1	0	39	39
Total		225	70	295

Chi-Square Tests

	Value	df	Asymptotic Significance (2-sided)	Exact Sig. (2-sided)	Exact Sig. (1-sided)
Pearson Chi-Square	144,455[a]	1	0,000		
Continuity Correction[b]	139,639	1	0,000		
Likelihood Ratio	134,302	1	0,000		
Fisher's Exact Test				0,000	0,000
Linear-by-Linear Association	143,965	1	0,000		
N of Valid Cases	295				

a. 0 cells (0,0%) have expected count less than 5. The minimum expected count is 9,25.

b. Computed only for a 2x2 table

Kaplan-Meier survival curves of: high mitotic count (> 10 per 10 HPF) versus mitotic count ≤ than 10 per 10 HPF in terms of PFS and OS (upper part of the figure, left and right side, respectively); and of high Ki67 value (> 20%) versus Ki67 lower than 20% in terms of both PFS and OS (lower part of the figure, left and right side, respectively). P-values have been considered as significant if < 0.05 and are reported according to the corresponding univariant COX regression analysis.

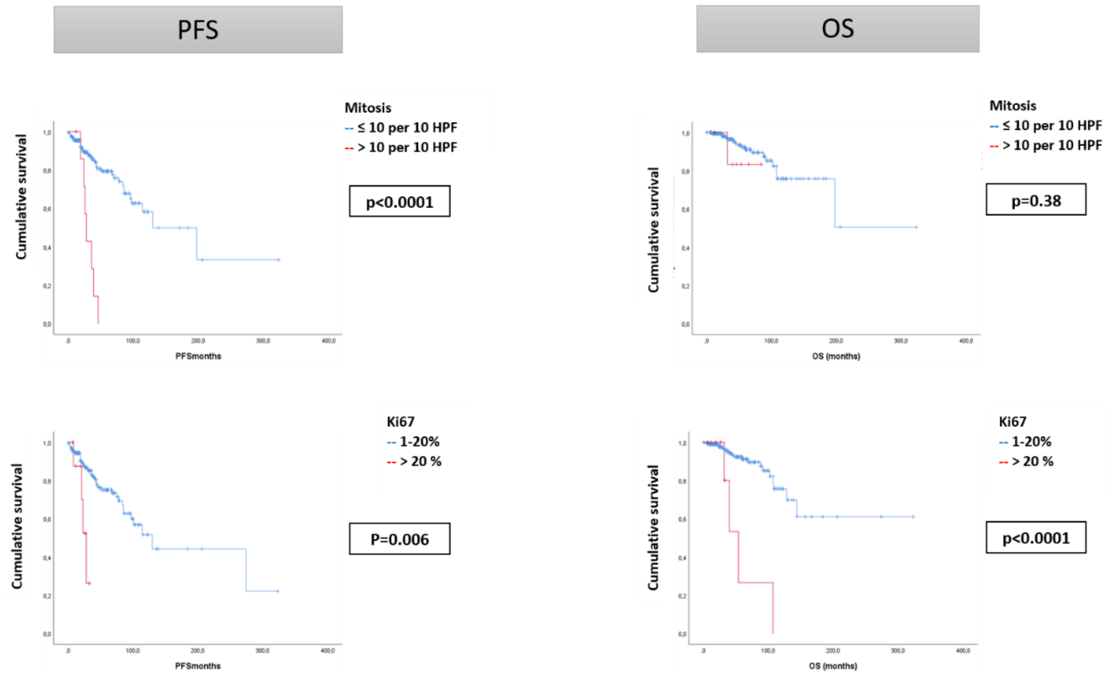

Supplement: Supplementary file 1 — Supplementary materials [file 12020_2022_3015_MOESM1_ESM.pdf]
